# Dietary Management of Eosinophilic Esophagitis: Tailoring the Approach

**DOI:** 10.3390/nu13051630

**Published:** 2021-05-12

**Authors:** Pierfrancesco Visaggi, Lucia Mariani, Veronica Pardi, Emma Maria Rosi, Camilla Pugno, Massimo Bellini, Fabiana Zingone, Matteo Ghisa, Elisa Marabotto, Edoardo G. Giannini, Vincenzo Savarino, Santino Marchi, Edoardo V. Savarino, Nicola de Bortoli

**Affiliations:** 1 Division of Gastroenterology, Department of Translational Research and New Technologies in Medicine and Surgery, University of Pisa, 56124 Pisa, Italy; pierfrancesco.visaggi@gmail.com (P.V.); marianilucia26@gmail.com (L.M.); v.pardi1@gmail.com (V.P.); emma.maria.rosi@gmail.com (E.M.R.); camilla.pugno@gmail.com (C.P.); massdimo.bellini@unipi.it (M.B.); s.marchi@med.unipi.it (S.M.); 2 Division of Gastroenterology, Department of Surgery, Oncology and Gastroenterology, University of Padua, 35128 Padua, Italy; fabiana.zingone@unipd.it (F.Z.); matteoghisa@yahoo.it (M.G.); edoardo.savarino@unipid.it (E.V.S.); 3 Division of Gastroenterology, Department of Internal Medicine (DiMI), University of Genoa, 16132 Genoa, Italy; emarabotto@libero.it (E.M.); egiannini@unige.it (E.G.G.); vsavarin@unige.it (V.S.); 4 Nutrafood, Interdipartimental Center for Nutraceutical Research and Healthy Food, University of Pisa, 56126 Pisa, Italy

**Keywords:** eosinophilic esophagitis, dietary management, six-food elimination diet, elemental diet, target elimination diet

## Abstract

Eosinophilic esophagitis (EoE) is a unique form of non-immunoglobulin E-mediated food allergy, restricted to the esophagus, characterized by esophageal eosinophil-predominant inflammation and dysfunction. The diagnosis requires an esophago-gastroduodenoscopy with esophageal biopsies demonstrating active eosinophilic inflammation with 15 or more eosinophils/high-power field, following the exclusion of alternative causes of eosinophilia. Food allergens trigger the disease, withdairy/milk, wheat/gluten, egg, soy/legumes, and seafood the most common. Therapeutic strategies comprise dietary restrictions, proton pump inhibitors, topical corticosteroids, biologic agents, and esophageal dilation when strictures are present. However, avoidance of trigger foods remains the only option targeting the cause, and not the effect, of the disease. Because EoE relapses when treatment is withdrawn, dietary therapy offers a long-term, drug-free alternative to patients who wish to remain off drugs and still be in remission. There are currently multiple dietary management strategies to choose from, each having its specific efficacy, advantages, and disadvantages that both clinicians and patients should acknowledge. In addition, dietary regimens should be tailored around each individual patient to increase the chance of tolerability and long-term adherence. In general, liquid elemental diets devoid of antigens and elimination diets restricting causative foods are valuable options. Designing diets on the basis of food allergy skin tests results is not reliable and should be avoided. This review summarizes the most recent knowledge regarding the clinical use of dietary measures in EoE. We discussed endpoints, rationale, advantages and disadvantages, and tailoring of diets, as well as currently available dietary regimens for EoE.

## 1. Introduction

Eosinophilic esophagitis (EoE) is a unique form of non-immunoglobulin E (IgE)-mediated food allergy, restricted to the esophagus, characterized by esophageal eosinophil-predominant inflammation and dysfunction [1,2,3]. It currently represents the most common eosinophilic gastrointestinal disorder as well as the most common cause of dysphagia and food impaction among children and young adults [4]. EoE was considered rare prior to the past three decades, but incidence rates up to 20 per 100,000 inhabitants annually have recently been reported [5]. However, EoE prevalence estimates vary with location. A significant increase in prevalence has recently been described in Europe and North America, where EoE now affects more than 1 in every 1000 people, including both children and adults [3,5]. In contrast, lower prevalence estimates have been reported in Japan and China, where EoE is diagnosed in 20 of every 100,000 esophago-gastroduodenoscopies (EGDS) [6]. Socially, EoE has a significant and negative impact on health-related quality of life (HRQoL), for both patients and caregivers [7]. Additionally, costs attributable to the disease are estimated to be of the same order of magnitude as hospital-related costs for inflammatory bowel diseases [7].

The pathogenesis of EoE lies within a complex network of disease contributors. Genetic susceptibility and environmental factors trigger cytokine pathways that drive chronic inflammation and progressive esophageal fibrosis [8,9]. Key genes that predispose patients to the development of the disease are involved in allergic inflammation and mucosal barrier integrity [10,11]. Esophageal inflammation is typically triggered by mucosal exposure to food antigens, but most patients are also sensitive to aeroallergens [12], which have been shown to induce seasonal exacerbations of esophageal eosinophilia [12,13]. Consistently, EoE is associated to other allergic conditions, including allergic rhinitis, asthma, eczema, and is believed to be a late manifestation of the atopic march [14]. 

The natural history of EoE is believed to be a progression [8], and clinical presentation varies with age and disease duration. Adults commonly present with dysphagia and bolus impaction, whereas children usually have reflux-like and non-specific upper gastrointestinal symptoms [3]. Esophageal chronic inflammation causes strictures, thickening, luminal narrowing, furrowing, transient and fixed rings formation [15], and is associated with esophageal dysmotility [16,17,18,19]. Of note, up to one fourth of patients lack endoscopic alterations [20]. 

The diagnosis requires an EGDS with esophageal biopsies demonstrating active eosinophilic inflammation with 15 or more eosinophils/high-power field (eos/hpf), following the exclusion of alternative causes of eosinophilia [1,2,21]. 

Therapeutic strategies comprise dietary regimens, proton pump inhibitors (PPIs), topical corticosteroids, biologic agents acting on molecular pathways of the inflammatory cascade, and esophageal dilation when strictures are present [3,21,22,23]. 

EoE virtually requires a lifelong therapy as it invariably relapses when treatment is withdrawn [3]. Avoidance of trigger food(s) offers a unique long-term, drug-free alternative for the management of the disease. Various dietary regimens are now available for patients with EoE who wish to remain asymptomatic without being on a pharmacological treatment. However, the choice depends on numerous patient-centered variables, as well as the healthcare setting [24]. 

This review summarizes the most recent knowledge regarding the clinical use of dietary measures in EoE. The following sections discuss treatment endpoints, rationale, advantages and disadvantages, and tailoring, as well as the currently available dietary regimens for EoE.

## 2. Literature Search

According to the aim of this narrative review we provided an overview of the evidence from original research articles, reviews, and clinical trials describing dietary treatments in patients with EoE. We conducted a literature review using the electronic databases PubMed, MEDLINE, and the Cochrane Library from inception to March 2021. We searched the medical literature using the term “eosinophilic esophagitis” and combined it using the set operator AND with studies identified with the following MeSH terms: treatment, management, diet, elimination diet, elemental diet, target diet, treatment endpoints. Two authors independently reviewed all manuscripts. All trial data was included as well other literature, based on a consensus decision of scientific merit by the reviewing authors. Restriction to studies written in English was applied. A manual search of the references listed in online database publications was performed to increase sources of information.

## 3. Therapeutic Endpoints in EoE

Dietary measures aim to induce remission of EoE. However, the definition of remission, therapeutic endpoints, and outcome measures is a matter of debate. Comparable to inflammatory bowel disease, EoE is a complex condition with clinical, endoscopic, and histologic markers of disease activity [25]. However, there may only be a modest correlation between these different outcomes, and the timeframe of response to treatment may vary between outcomes [25,26,27]. Recently, a framework to evaluate treatment outcomes in EoE has been proposed (Table 1) [25]. 

The primary goal of the treatment of EoE is to control esophageal eosinophilia and inflammation. According to the latest guidelines [21], this corresponds to a persistent reduction of eosinophils to <15 eos/hpf in esophageal biopsies. However, a recent systematic review documented a high variability in the definition of histologic remission in studies regarding the treatment of EoE, ranging from 0 to ≤20 eos/hpf [28].

Patient-reported outcome measures have been proposed as co-primary therapeutic outcomes in EoE [29,30], and patient-centered questionnaires have been developed for clinical use. These include the Eosinophilic Esophagitis Activity Index, the Dysphagia Symptom Questionnaire for adults, the Pediatric Quality of Life Inventory EoE, and the Pediatric EoE Symptom Score for children [29]. Of note, there is a considerable mismatch between symptoms and endoscopic and histological findings [26]. Therefore, resolution of symptoms alone is inadequate to assess the biological activity of EoE [31].

Endoscopic findings also reflect the severity of the disease, and their improvement should be included among the goals of treatment. The endoscopic reference score by Hirano et al. (EREFS) [32] reduces variability among endoscopists in the assessment of disease activity and is reproducible. In particular, the EREFS scores the five major endoscopic featuresof EoE, namely exudates, rings, edema, furrows, and strictures, as well as minor findings, including felinization, narrowed caliber, and crêpe-paper esophagus.

## 4. Rationale for Avoidance Strategies in EoE: Food Is the Trigger

When esophageal eosinophilia was still considered a consequence of the exposure to acid gastro-esophageal reflux [33], Kelly et al. [34] introduced the concept that food allergens trigger EoE. In 1995, a seminal report documented that 10 children with esophageal eosinophilia refractory to PPI, antacids, and fundoplication, were fed exclusively with elemental formulas devoid of allergenic capacity. The amino-acidic dietary regimen led to clinical and histological remission in eight patients, and to significant improvement in two, establishing the etiologic role of food antigens in EoE. However, gastroesophageal reflux disease also plays an important role in the pathogenesis of EoE, by favoring esophageal mucosal impairment [35].

In 2006, Kagalwalla et al. [36] demonstrated that removing the most common foods related to food allergy from the diet of 35 children for six weeks induced remission of esophageal eosinophilic inflammation in up to 74% of patients. These findings have been further confirmed both in children and adults [37,38,39]. Therefore, it was clear that food allergens have a causative role in EoE following Koch’s postulate, as demonstrated by resolution of inflammation once the food is avoided and recrudescence upon reintroduction [40].

The exact mechanism leading to esophageal inflammation in EoE is not completely understood yet. Both innate and adaptive immune response are involved in esophageal inflammation, but delayed cell-mediated hypersensitivity is predominant [9]. As opposed to food allergy, the role of IgE is marginal in EoE, and Th-2 lymphocytes seem to play a key role [3,9]. Consistent with this, lymphocyte-deficient mice cannot develop EoE, but IgE-deficient mice can [41,42]. Specific IgG4s offood antigens may be found to be elevated in patients with EoE howevertheir pathogenetic role is still unclear, as they could be an attempt of the immune system to down-regulate the Th-2 response, or a class switch from IgE in the natural history of the disease [3,21,40,43].

## 5. Balancing Pros and Cons of Dietary Management

Dietary regimens represent a long-term and effective treatment for EoE, comparable to topical steroids and PPI (Table 2) [22,30,44]. However, both clinicians and patients should acknowledge the benefits and drawbacks of this strategy (Table 3). 

Most important of all, diets allow for the identification of individual trigger food(s) and avoid virtually lifelong pharmacological treatments and associated adverse events. Even large food deprivations seem not to worsen the nutritional status or cause growth deceleration in children [46]. However, long-term dietary avoidance has the potential to cause nutritional imbalances, similar to as has been described for food allergies [50,51]. Up to one third of children with EoE may present with failure to thrive secondary to malnutrition [33]. In this setting, an assessment, and further follow-ups by a dietician for additional nutritional planning for provision of adequate calories, appropriate substitutes foods, and supplements, are of paramount importance [24,52]. Switching from a normal diet to an elimination diet associated with elemental formulas may also enhance spontaneous oral intake in infants [1]. On the other hand, children feeding with liquid formulas (i.e., an elemental diet) do not engage masticatory muscles and are at increased risk of delayed onset of oral-motor skills [47].

Patients following a dietary regimen for EoE should be aware of the necessity of repeated follow-up endoscopies. After the initial food elimination diet, an EGD after 6 to 12 weeks is necessary to show that the chosen diet has induced histologic remission [21]. Once remission is achieved, foods or food groups are sequentially reintroduced to identify the trigger food(s). After each reintroduction, an EGD is performed in between 6–12 weeks to confirm or exclude remission, before proceeding to the following food [2,21,31].

The need for strict avoidance of all kinds of table foods impacts over HRQoL in patients with EoE, causing social and psychosocial deterioration [48]. The social cost of EoE burdens both patients and other family members. High motivation to diet rigorously is required by the patients, and much effort needs to be in place to ensure safe foods are available at all times, including when participating in community events [24]. From a financial perspective, diets are cost-effective based on the cost of medical procedures and prescriptions [49]. For patients, adhering to an elimination diet may carry an excess annual cost of greater than $650–720 compared to an unrestricted diet, associated with the logistical burden of visiting specialty grocery stores to buy foods unavailable at standard stores [53].

## 6. Tailoring the Diet to the Patient and Available Resources

Before initiating an elimination diet, a shared agreement between the clinician and the patient and/or patient’s family is necessary to identify which approach is clinically most appropriate and practically most viable. Multiple variables influence the appropriateness of a dietary regimen, including the patient’s preferences and characteristics, and availability of facilities for adequate follow-up.

First and foremost, patients should be given thorough information regarding the advantages and disadvantages of diets, so to choose judiciously. From the physician’s perspective, individual characteristics of candidates should be accurately considered before proposing an elimination diet. Although growth failure and feeding difficulties are not a contraindication for a dietary elimination therapy [54], a comprehensive assessment of pre-treatment diet, nutritional status, and possible feeding difficulties is necessary to establish whether a diet is feasible [24].

Up to 67% of patients with EoE have IgE-mediated food allergies, and 2% have celiac disease [55]. Such patients should be discouraged from undertaking elimination diets requiring additional restrictions that would hamper adherence and compromise HRQoL [33]. Similarly, patients who have more than four foods triggering the disease are not best candidates for dietary measures, as large restrictions would be necessary [39,56].

Patients and family members should be aware of the increase in weekly shopping costs and acknowledge the logistical complexity associated with the supply of foods only available at specialty stores [53]. Additionally, eating habits, ability to cook, necessity to eat out of the house, and reliance on pre-prepared foods should be considered prior to opting for a dietary treatment [40].

Candidates to dietary therapy should also be ready to undergo multiple endoscopies for the assessment of efficacy [2,21]. Finally, aspects related to the healthcare center where the follow-up is performed should be taken into consideration. The facility must guarantee EGDSs for re-assessment after food eliminationscheduled 6–12 weeks apart and be able to offer endoscopic procedures within a shorter time frame in case of recurrence upon food reintroduction [33].

## 7. Diets

The final goal of the diet is to identify which foods activate EoE in each individual patient. The process is intended to customize a dietary regimen in which only the culprit foods are restricted. Currently available strategies include elemental diets (EDs) and empirical or target elimination diets. 

### 7.1. Elemental Diet

In EDs all kind of foods are replaced with liquid formulations containing single amino acids, carbohydrates, and medium-chain fatty acids devoid of any antigenic capacity [31,43]. Since their prime time in EoE [34], several studies have repeatedly proven the efficacy of EDs in inducing and maintaining clinical and endoscopic remission in both children and adults. Most studies have enrolled children [36,37,57,58,59,60,61], consistently demonstrating high success rates close to 90%. Fewer studies have included adults. Peterson et al. [62] prospectively enrolled adult patients with EoE undergoing exclusive nutrition with elemental formulas for four weeks. According to the per protocol analysis, the overall efficacy was 94.4% in terms of histological remission, but it was reduced to only 58.6% in the intention-to-treat analysis due to considerable non-adherence to the diet [62]. In the study of Warners et al. [63], adults showed good adherence to the diet for four weeks with histological remission in 71% of cases. Finally, a systematic review with meta-analysis confirmed that elemental diets can achieve histological remission in up to 90.8% of patients of all ages [44].

Despite impressive remission rates, amino-acidic based diets have significant inconveniences that hamper their use in daily life. Although flavored or unflavored formulations are currently available, elemental formulas lack palatability and many patients may not accept their taste. Accordingly, up to 80% of children and adolescents feeding with elemental formulas may require a nasogastric tube for nutrition [57]. Beyond infancy, elemental diets have a worse therapeutic index than all the other available treatment options [40], and adults frequently fail adherence to diet within 2–4 weeks [62,63]. 

Due to scarce compliance, cumbersome administration, and high costs, exclusive EDs are unfeasible in the long term. However, they may have a role in carefully selected cases. EDs may represent a rescue therapy for those who wish to remain in remission while investigating the cause of their disease [64], or a bridge therapy for those waiting for investigational drugs [21,33]. In extreme cases, EDs may be administered to induce quick remission in infants with a limited intake of solid foods, before proceeding to a controlled food reintroduction to discover EoE triggers [40].

### 7.2. Empiric Elimination Diets

Empiric elimination diets are therapeutic interventions in which the most common foods known to activate EoE are excluded from the diet. The restriction is based on the prevalence of triggers in the population, and primarily includes milk/dairy, wheat/gluten, egg, soy, nuts, and seafood [34,37,38,39,54,56,59,65,66,67,68,69]. Restricting this group of six foods from the diet is known as six-food elimination diet (SFED). According to a meta-analysis, the overall effectiveness of SFED to induce remission of EoE was 72% (95% CI, 66–78%), with extreme homogenous results in children and adults (I^2^statistic = 0%) [44]. In general, the wider the dietary restriction, the likelier the patient is to respond to the elimination diet and show clinical and histological improvement [40]. Accordingly, in a retrospective study comparing different types of elimination diets, Spergel et al. [65] documented a response rate of 77% with an eight-food elimination diet, in which milk, egg, soy, wheat, chicken, turkey, beef, and pork were restricted.

Once remission is achieved, single foods or groups of foods should be sequentially reintroduced, and an EGD with at least six esophageal biopsies should be performed between 6–12 weeks from the challenge to assess if histological remission persists [1,2,21,31]. According to studies on SFED conducted to date, fish/shellfish, nuts, and soy/legumes have the lowest prevalence among EoE triggers, withmilk, egg, and wheat being the most common [34,37,38,39,54,56,59,65,66,67,68,69]. Food reintroduction could start from the least allergenic foods and proceed to the most or vice versa [45,70,71] and may be performed singly or in groups. For high-risk foods (milk, wheat, soy, and egg) it is preferable to reintroduce the foods one at a time, whereas medium-risk foods (legumes, seafood, nuts) may be re-administered at one time, and low-risk foods (fruit and vegetables) may reintroduced in groups [40]. Variations in the foods included in the SFED have been reported, including removing shellfish additionally to fish [39], all legumes instead of soy, and all grains including rice, corn, and all gluten-containing cereals instead of wheat [38]. The clinical relevance of these small differences seems negligible and does not hinder the outcome of the diet [33]. Importantly, children with a present or previous history of an IgE-mediated food allergy should be referred to an allergist before being commenced on alimentary restrictions and reintroductions [40]. The reason is that children who have serum food-specific IgE but tolerate the food due to chronic exposure, may develop IgE-mediated hypersensitivity and anaphylaxis upon reintroduction following prolonged avoidance [72,73].

The whole restriction-reintroduction process aims to identify the trigger food(s), as maintenance of remission in the longterm should be done by exclusively avoiding foods that sustain inflammation in the individual patient [74]. When provocative aliments are discovered and avoided, sustained remission is achievable. Several studies confirm that adults strictly avoiding their trigger foods can be maintained on clinical and histological remission for up to three years [33,38,39,66,75].

Following the earliest report of a SFED as a treatment for EoE [36], multiple approaches have been proposed to optimize resources and outcomes in the diagnostic-therapeutic work-up of elimination diets. A summary of the currently available food elimination strategies follows.

#### 7.2.1. SFED: The “Classic” Top-Down Approach

Classically, a SFED starts with a large restriction of foods, followed by gradual reintroduction. This approach is well established and is known as top-down SFED. Patients are instructed to avoid milk, wheat, egg, soy, nuts, and seafood for at least six weeks. Subsequently, each responder to a SFED can only be identified through individual food reintroduction, followed by histological re-evaluation [3,45]. Specific food triggers identified by sequential food reintroduction challenge have been mostly cow’s milk, wheat, egg, and soy/legumes [38,39,68]. A meta-analysis documented the high effectiveness of a SFED both in children (72.8%) and adults (71.3%) [44]. However, the compliance to large food restrictions is scarce in the long term [62]. Additionally, up to 85% of patients undergoing a SFED have been found to have only one to two trigger foods after undergoing six food challenges and six endoscopic procedures [43,56]. These findings have paved the way for less restrictive and more resource-saving strategies.

#### 7.2.2. Four-FoodElimination Diet

A four-food elimination diet (FFED) restricts dairy, wheat, egg, and legumes [21]. The rationale for a FFED is to make the diet more tolerable for patients, and to avoid unnecessary endoscopies. Two multicenter prospective non-randomized trials in adults and children have assessed the clinical feasibility of this approach. As regard to adults [56], 67% of PPI non-responders achieved clinical remission, as defined by a reduction of 50% of baseline Dysphagia Symptoms Score, and 54% achieved clinic-histologic remission following six weeks of avoidance of dairy products, wheat, egg, and legumes. Noteworthy, milk and wheat were triggers of EoE in 50 and 31% of cases, respectively [56]. In children who underwent a FFED restricting cow’s milk, wheat, egg, and soy for eight weeks, remission was achieved in 64% of cases [54]. After food reintroduction, 85% of flare-ups were triggered by cow’s milk, and 33% by wheat.

#### 7.2.3. The Step-Up Approach

**Figure 1:**
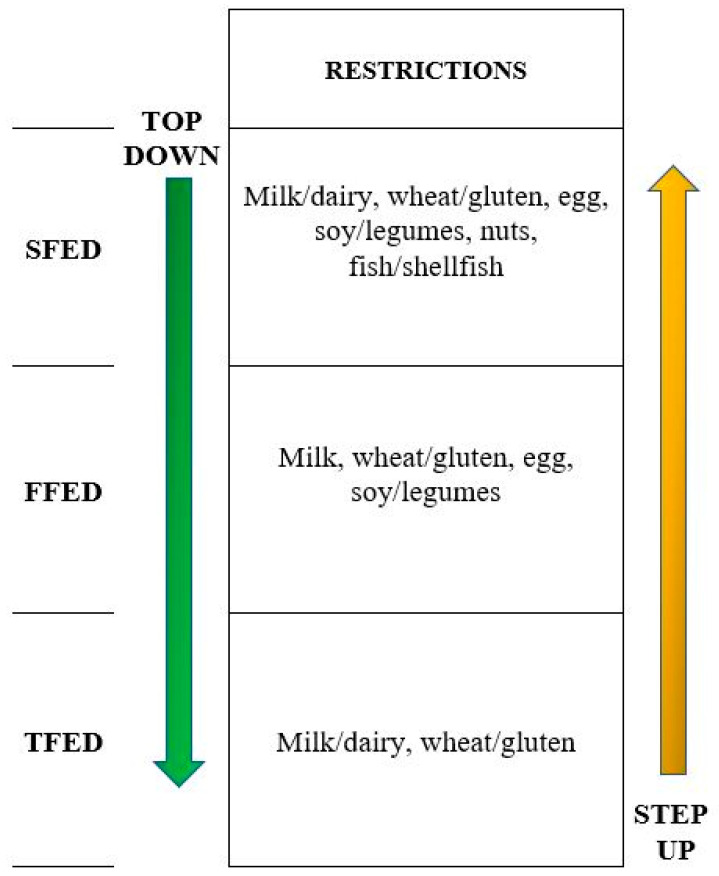
Top-down and step-up strategies in elimination diets [45]. SFED: six-food elimination diet; FFED: four-food elimination diet; TFED: two-food elimination diet.

The large number of dietary restrictions and endoscopic procedures used in elimination diets are considered to be the best deterrents for patients and physicians. However, most patients with EoE have just one or two trigger foods, and part of the therapeutic-diagnostic work-up may be unnecessary. Because milk and wheat are the most common foods that trigger EoE at all ages [34,37,38,39,54,56,59,65,66,67,68,69], a recent multicenter prospective study enrolled children and adults to assess the effectiveness of a step-up elimination diet, starting from the less restrictive diet to the most (Figure 1) [45]. Patients with EoE who did not respond to an eight-week course of PPI were instructed to adhere to a two-food group elimination diet (TFED), thus restricting all dairy products and gluten containing cereals. After six weeks of diet, 43% of patients were in remission. Non-responders to a TFED were offered to step-up to a FFED, which achieved remission in 17% of cases. Those whose EoE was still active following six weeks of the FFED eventually underwent a SFED, which could offer remission to 19% of patients. Cumulative per-protocol clinic-histologic remission rates after TFED, FFED, and SFED were 43%, 60%, and 79%, respectively. This revised step-up approach, compared to a classic top-down SFED, could reduce the diagnostic time and the number of endoscopies by up to 30%. 

#### 7.2.4. One-Food Elimination Diets

Studies evaluating elimination diets have consistently proven that milk/dairy and wheat represent the most common trigger foods in patients with EoE. Additionally, up to 70% of patients responsive to a TFED have only one trigger, with milk being the most common [45]. Accordingly, several studies have been designed to assess the efficacy of a one-food elimination diet (OFED) in which either only milk or wheat/gluten were restricted [58,76,77,78,79]. In a meta-analysis the overall efficacy of a milk-free diet was 68.2% (95% CI, 47.8–85.6), whereas gluten-free diets achieved remission in 58.7% of patients (95% CI, 23.1–89.7) [44]. More recently, a retrospective study documented that children adhering to a dairy-free diet could achieve remission in 56.9% of cases [80]. However, most of the patients had been taking PPIs as an add-on to the dietary regimen. Two other recent retrospective studies reported a variable efficacy of 25–58% of dairy-free diets [78]. Finally, two clinical trials aiming to compare the effectiveness of an OFED restricting milk over a SFED in adults (NCT02778867), and over a FFED in children (NCT02778867), recently completed enrolment, but their results are not yet available as a full paper. 

### 7.3. Target Elimination Diet

The philosophy of target elimination diets is to recognize and restrict individual trigger foods on the basis of food allergy tests results, avoiding empirical eliminations. In this setting, the utility of skin prick tests (SPTs) and atopy patch tests (APTs) has been explored with unsatisfactory results. SPTs reveal immediate IgE-mediated allergic reactions [81], whereas APTs assess delayed non-IgE, cell-mediated reactions [82]. Early studies have reported conflicting results of selective elimination diets based on skin testing. In children, dietary avoidance based on the results of SPTs and serum IgE to foods demonstrated a lack of response [24]. In another large series of children, dietary restrictions guided by the combination of SPT and APT results led to 75% symptomatic and histologic improvements, with a positive predictive value (PPV) of 74%, and a negative predictive value (NPV) of 88–100% for almost all foods [83,84]. As regard adults, considerably lower response rates of 26–35% have been reported [67,85]. A meta-analysis including 14 studies on combinations of SPTs, APTs, and serum-specific IgE to food-based elimination diets documented a low overall efficacy of 45.5% (95% CI, 35.4–55.7%) across all ages [44]. However, the efficacy of skin tests in children was significantly greater than in adults (47.9% versus 32.2%). Noteworthy, the performance of skin allergy tests for the prediction of trigger foods in EoE had considerable variability in the response rate (I^2^statistic = 75.1%), indicating low reproducibility [44]. The sensitivity and specificity of SPTs to identify trigger foods in EoE has been demonstrated as consistently low with PPVs ranging from 26.3–83.3%, and NPVs of 30% for cow’s milk, and 79–90% for egg, wheat, and soy [33,46]. APTs demonstrated similar results, with PPVs of 12–86%, and NPVs of 40, 56, and 67% for milk, egg, and wheat, respectively [46,65].

These findings suggest that the diagnostic accuracy and reproducibility of food allergy skin tests is insufficient to design effective diets for EoE patients, thus their use in the clinic should be abandoned [3,21,24,33].

## 8. Discussion and Conclusions

Avoidance of trigger foods remains the only non-medical option to prevent esophageal chronic inflammation in patients with EoE. Dietary interventions target the cause of the disease and have comparable efficacy to pharmacological treatments, which can only act once inflammation has already occurred. EoE is a chronic condition that requires long-term treatment to prevent recurrence [3]. Accordingly, discussion of long-term management and long-term endurance on the diet needs to be addressed early when proposing alimentary restrictions to patients [24]. The dietary regimen must be tailored around patients’ preferences and characteristics. Additionally, the availability of resources for punctual follow-ups should be ensured. Multiple EGDSs are needed during a dietary treatment to establish the efficacy of a diet, reintroduce foods, and monitor the long-term efficacy of the diet and food tolerance [40]. Therefore, the best candidates for dietary restrictions are strongly motivated patients who wish to remain off drugs and still achieve remission [33]. Patients should acknowledge the drawbacks of avoidance strategies, including a significant impact on HRQoL and an increase of weekly shopping costs compared to unrestricted diets [53]. Especially for pediatric patients, the inclusion of a nutritionist for assessment of diet feasibility and nutritional follow-up is recommended [52]. Additionally, an allergist should be included in the diagnostic-therapeutic work-up of patients with present, past, or possible history of an IgE-mediated food allergy, as prolonged avoidance of specific foods during restriction periods may cause anaphylaxis upon reintroduction in those who are predisposed [72,73].

There are currently several dietary management strategies to choose from, each having its specific efficacy, advantages, and disadvantages. EDs represent the most effective treatment for EoE, having 90.8% efficacy at all ages [44]. However, burdensomeness and high costs make EDs only suitable for short-term treatments for selected patients. Elimination diets with multiple possible approaches (top-down, step-up, OFED) are adequate and effective long-term maintenance strategies, especially for those who have one or two trigger foods, in whom only few restrictions would be sufficient for disease control [45]. SPTs, APTs, and food-specific IgE results should not guide dietary restrictions in patients with EoE because they lack reproducibility and predictive value [2,21,44]. Interestingly, the possibility to evaluate esophageal sensitization through esophageal prick tests has recently been described [86]. The local injection of allergens into the esophageal mucosa detected abnormal responses to food allergens (complete luminal obstruction, blanching of the mucosa, erythematous wheals) in patients with EoE, correlating, although still imperfectly, with culprit foods. Further studies are needed to assess whether allergy testing may still have a place in the dietary management of EoE.

## Figures and Tables

**Table 1:** Therapeutic endpoints and outcome measures in EoE [25].

	Outcome Measures	Histology	Symptoms	Endoscopy
Treatment Response				
**Normalization**		<1 eos/hpf ^a^	Decrease >90% in symptoms score *	Resolution of endoscopic findings, EREFS ^b^ < 2
**Response**		14 -1 eos/hpf	Decrease 30–90% in symptoms score	Improvement of endoscopic findings, EREFS ≥ 2
**Non-Response**		≥15 eos/hpf	Decrease < 30% in symptoms score	Persistence of endoscopic findings similar to baseline

^a^ Eosinophils/high-power field; ^b^ Endoscopic reference score; * The same validated symptom score should be used at baseline and at following assessments.

**Table 2:** Efficacy of dietary approaches for inducing histologic remission in eosinophilic esophagitis [44,45].

Type of Diet	Adults, % (95% CI)	Children, % (95% CI)	Overall, % (95% CI)
**Elemental**	94.4	90.4 (83.5–95.5)	90.8 (84.7–95.5)
**SFED**	71.3 (61.7–80)	72.8 (62.5–82)	72.1 (65.8–78.1)
**FFED**	46.2	60	53.4 (35.7–70.6)
**TFED**	44	40	43
**Gluten-free**	88.8 (50.5–99.1)	45.5 (2.6–93.8)	58.7 (23.1–89.7)
**Milk-free**	100	66.3 (44.7–84.8)	68.2 (47.8–85.6)
**Target**	32.2 (17.8–48.7)	47.9 (36.8–59.1)	45.5 (35.4–55.7)

SFED: six-food elimination diet; FFED: four-food elimination diet; TFED: two-food elimination diet.

**Table 3:** Advantages and disadvantages of dietary regimens [1,2,21,24,46,47,48,49].

Advantages of Diets	Disadvantages of Diets
✓ Effective in inducing remission ✓ Long-term strategy ✓ Cost-effectiveness ✓ Do not cause nutritional imbalances or growth deceleration in children * ✓ Allows the identification of trigger food(s) ✓ Allow the avoidance of long-term courses of steroids or PPIs ^a^ ✓ May favor spontaneous oral intake in children	o Assessment of efficacy requires repeated endoscopies o Adherence requires strong motivation o Negative impact over HRQoL ^b^ o Risk of delayed onset of oral motor skills ^Ŧ^ o Higher costs than unrestricted diet o Supply of suitable foods may be cumbersome

^a^ Proton pump inhibitors; ^b^ Health-related quality of life; * According to follow-ups up to two years [46]; ^Ŧ^ In small children feeding exclusively with liquid elemental formulas [47].

## Data Availability

No additional data available.
